# A “Light Bulb Moment” in Understanding Public Health for Undergraduate Students: Evaluation of the Experiential “This Is Public Health” Photo Essay Task

**DOI:** 10.3389/fpubh.2017.00116

**Published:** 2017-05-22

**Authors:** Kate Joanne Dundas, Vibeke Hansen, Suzanne Outram, Erica L. James

**Affiliations:** ^1^School Medicine and Public Health, University of Newcastle, Newcastle, NSW, Australia; ^2^University Centre for Rural Health (North Coast), Sydney School of Public Health, The University of Sydney, Lismore, NSW, Australia

**Keywords:** teaching, public health, undergraduate, assessment, experiential learning, curriculum

## Abstract

A lack of understanding of the importance of public health both within the community and in the tertiary education setting is a significant impediment to improvement in population health. The international campaign “This is Public Health” (TIPH) has been promoted widely as a strategy to increase community awareness and attract and inspire the next generation of public health professionals. This paper describes and evaluates student perceptions of a TIPH photo essay and reflective task in order to explore the pedagogical and learning outcomes related to undergraduate students’ public health knowledge. The aim of the analysis was to understand (1) if the task led to increased awareness of public health, and if so, the process of how an understanding of public health develops, and (2) how the interactive nature of the experiential TIPH task leads to depth of understanding.

**Methods:**

This study was undertaken at the University of Newcastle (UON), NSW, Australia. A qualitative study design using a descriptive case study methodology was employed. One-hundred and thirty-nine undergraduate students taking part in a semester-long, introductory public health course provided informed consent and completed a TIPH photo essay and reflective task as a compulsory assessment. Analysis of the student reflections was performed using a general inductive approach to qualitative thematic analysis.

**Results and discussion:**

Analysis of the reflections indicated that completion of the photo essay and reflective task revealed two strong thematic clusters each with a number of subthemes. The most important findings were the six strong data clusters around students’ new and deeper understanding of Public Health. Additionally, four separate data clusters around the pedagogy of the task were revealed. The task also impacted beyond knowledge improvement and academic performance. Students alluded to an increased appreciation of their own health, a new recognition of the importance of preventative health measures, and an improved analytical awareness of health determinants and the measures in place to protect health.

**Conclusion:**

The TIPH photo essay and reflective task was successful in providing undergraduate students with an experiential activity that resulted in increased knowledge and understanding of public health strategies. The task was a valuable pedagogical experience.

## Introduction

Public health initiatives are responsible for the significant increase in life expectancy in the last 100 years, yet most community members have little understanding of what public health actually is (1). This lack of understanding of the importance of public health within the community is a significant impediment to improvement in population health (2, 3) and a substantial road block when teaching undergraduate students who struggle to understand its elusive nature (4). Developing solid pedagogy to ensure tertiary students have a rich, meaningful understanding of the nature and scope of public health is particularly challenging (5). This requires a reorientation of curative or biomedical approaches to health and undergraduate education, to that of wider public health initiatives. It also emphasizes the importance of liberal education and the need to build a solid foundation of knowledge and understanding of public health across all disciplines (6, 7).

There is a growing recognition of the value of public health education for undergraduate students (8, 9). This requires teaching and learning strategies designed to enhance students’ understanding of, and engagement with, the study of public health. These strategies must be appropriate for an undergraduate audience. One of the key recommendations for providing public health education to all undergraduate students is that these courses include experiential/active learning and/or a service-learning task to engage students and involve the community (3, 9). It is widely accepted that experiential learning is the “gold standard” in secondary and tertiary education, particularly in response to increased understanding of learning theories and cognitive development (10). Passive learning (i.e., receiving information in traditional didactic approaches) does not result in meaningful learning and thinking (11, 12), while engaging students in the learning process (experiential learning or active learning) leads to more active learning and better outcomes (12). In public health education, an example of passive learning might be to tell a student directly (didactic approach) that non-smoking signs and warnings on cigarette packets are interventions to reduce tobacco-related illness. In contrast, an example of experiential (active) learning might require students to research, identify, locate, and even photograph a variety of interventions that reduce tobacco-related illness in a community. The goal being to enable the student to understand in-depth the nature and scope of public health as an approach to reducing tobacco-related illness from a population wide perspective. The student will develop an understanding of how a country addresses tobacco-related illness with a broad spectrum of legislation and interventions. This depth of understanding can then assist students apply this knowledge to analyze other population health problems (such as alcohol misuse or obesity). The student builds on the previously learned concepts, which leads to mastery.

As the name suggests, the “experience” is intrinsic to true understanding of a concept. However, having an “experience” as a sole learning activity does not always ensure learning will occur. Kolb’s model for experiential learning outlines three essential elements that must be satisfied for deep, meaningful understanding to occur: (1) an experience; (2) a reflection (or contemplation); and (3) application (or conceptualization). These elements remain in a “recurring cycle” with hypotheses and impressions of the experience constantly being broadened and expanded (13). Thus, reflection, consciously thinking about or analyzing past or present events, actions, or processes (14), is an essential component of the learning process. “*Reflection is a powerful mechanism behind learning. We do not learn from experience* … *we learn from reflecting on experience*” Dewey (15) as cited in Ref. (16). Di Stefano et al. explained that in higher education, reflection allows the learner to synthesize, abstract, and articulate key lessons taught by experience. It is this process that defines lifelong learning, which is especially important for the development and continuous improvement of professionals (17).

**Figure 1:**
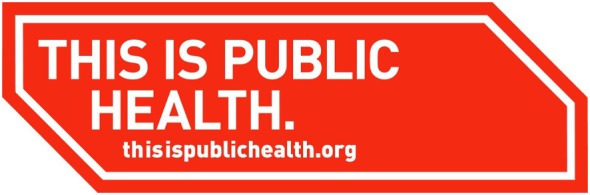
**“This is Public Health” campaign red sticker/label**.

The Association of Schools of Public Health (ASPH) in the USA pioneered one such dynamic experiential initiative that utilizes “branding” of public health as the main strategy. It was developed to help students link with communities in understanding the true meaning and scope of public health. Based around New York’s “urban skate culture,” the initiative formed the basis of a campaign that used the concept of “tagging” in an effort to engage and reach younger generations (18). Red (removable) stickers labeled “This is public health” (TIPH) (Figure 1) are used to “tag” public health interventions. The stickers have been utilized in many ways across high schools, universities, area health services, community health centers, local government, and individuals worldwide, and various approaches utilizing the campaign in tertiary education have been well received (18, 19). The TIPH campaign has a strong presence on Pinterest, Facebook, Flickr, Instagram, Twitter, and Google maps. However, a review of research literature revealed few and conflicting formal evaluations of the TIPH activity in tertiary education settings. One study in 2009 evaluated a pilot recycling program (19) at the University of South Carolina. Using the TIPH campaign as the platform, the study revealed a significant increase in awareness and effort of recycling. Another study used the campaign to evaluate undergraduate student knowledge and understanding of public health in an Australian university (4). Results of the latter study indicated that students perceived a higher level of their own knowledge and understanding of public health than was reflected in their assessment tasks even after utilizing the TIPH campaign.

The University of Newcastle (UON) has been a participant in the TIPH campaign since 2008 (3) using it as the basis of a photo essay task. In recognition of the importance of reflection as part of the experiential learning activity, the TIPH photo essay task at UON also incorporates a 500-word reflection. The TIPH photo essay task and reflection is utilized across three large undergraduate courses that are core components of several health and education programs. The current study set out to evaluate the role of the interactive TIPH photo essay and reflection task in improving and deepening undergraduate students’ understanding of public health and awareness of its breadth of meaning and to explore the subjective experience of gaining this understanding. The aim of the study was to understand (1) if the task led to increased awareness of public health, and if so, the process of how an understanding of public health develops, and (2) how the interactive nature of the experiential TIPH task leads to depth of understanding.

## Materials and Methods

### Study Design

In order to examine in detail the process and outcomes of this practical and reflection-based learning experience, a qualitative study design using a descriptive case study methodology was employed (20). Such a design allows focus on a unique cohort of students exposed to the same learning experience and offers a means of exploring complex social units influenced by multiple contextual variables, while acknowledging the diverse academic paths of the students (20).

### Sample and Recruitment

Potential participants in this study were students (*n* = 210) taking part in a semester-long introductory public health subject at UON, NSW, Australia. Students were enrolled in a range of allied health courses including physiotherapy, occupational therapy, medical radiation science, podiatry, nutrition and dietetics, biomedical science, psychology, oral health, and others. All enrolled students were required to complete the TIPH photo essay and reflection task; however, only data from those students who provided written informed consent were included in the current analysis. The University of Newcastle Human Research Ethics Committee approved the study (H-2010-0024). All participants gave written informed consent in accordance with the Declaration of Helsinki.

### Procedure

A specific assessment task based on the TIPH campaign and designed to promote student learning through hands-on experiences and reflection was devised. The task required students to:

1. Identify 10 examples of public health in their community, and photograph each with the temporarily affixed TIPH sticker. Affixing the sticker ensured that students did not simply obtain images from the internet.
2. For each photograph, describe the public health issue in detail, including the implications of the issue, affected populations, identified stakeholders and burdens to society as well as existing intervention strategies in place to address the issue.
3. Complete a 500-word reflection task designed to facilitate a deeper understanding and interpretation of their learning experience. Gibbs’ (21) reflective cycle was introduced to students as a means of providing a framework and systematic approach to using reflection as a means of enhancing learning. The purpose of this reflection was to constitute a personal account of the learning which took place, including the student’s feelings and judgments relating to the experience, analysis of their strengths and weaknesses, as well as an evaluation of their learning goals for the future.

### Analysis

A computer program (NVIVO 10) was used to assist with the organizational aspects of data analysis. Analysis was conducted by the author Vibeke Hansen with identified emergent themes discussed with a second author (Kate Dundas) to check for meaning and achieve consensus. Analysis was performed using a general inductive approach to qualitative thematic analysis (22). Initially, a hierarchical coding scheme was developed based on a thorough review of the complete data set. During a subsequent coding cycle, the coding scheme was further revised and expanded, while further inductively derived codes were formulated from meaning units arising from the data. Following coding of all the student reflections, emerging themes and relationships in the data were identified. Further review of the content within these emerging themes, and their fit to the entire data set, was conducted as part of a final refinement of the themes. These were then defined and formed the basis of the analytic narrative presented in this paper, which explores the pedagogical and learning outcomes related to students’ public health knowledge and general academic development.

## Results

The TIPH photo essay and reflection task was completed by all 210 students enrolled in the course in 2010, of which 139 (66%) consented to their submissions being utilized in the current analysis. Consenting students ranged from first to fourth year University students, with the majority being in their first year of an allied health course (e.g., physiotherapy, medical radiation science; Table 1).

**Table 1:** **Demographic characteristics of participants (*n* = 139)**.

	*n* (%)
**Gender**	
Male	53 (38)
Female	68 (62)
**Program enrollment**	
Physiotherapy	58 (42)
Occupational therapy	32 (23)
Nutrition and dietetics	24 (17)
Medical radiation science	14 (10)
Biomedical science	8 (6)
Psychology	3 (2)
**Year of study**	
First year	102 (73)
Second or later	37 (27)

**Figure 2:**
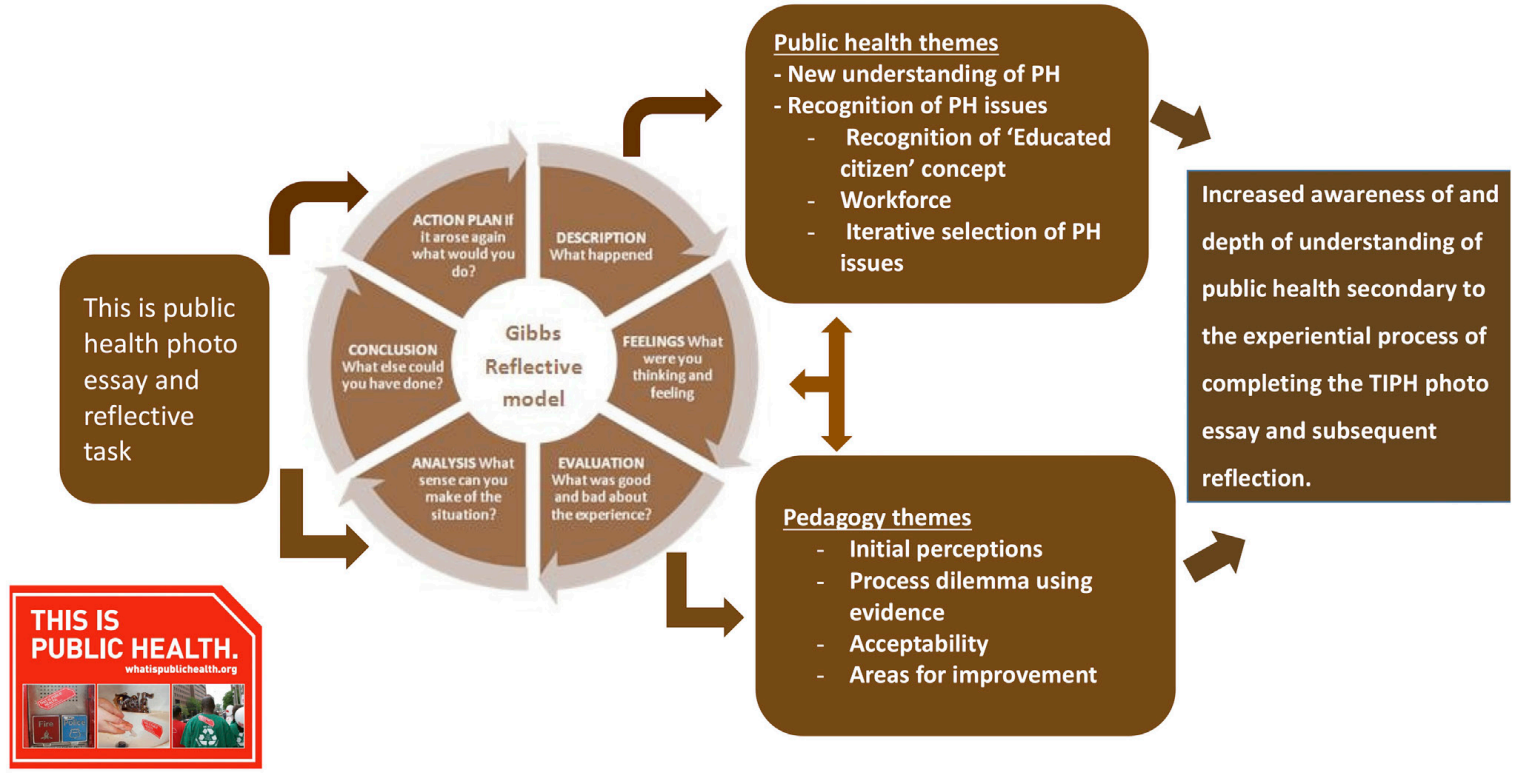
**Thematic analysis conceptual framework**.

The thematic analysis revealed six data clusters relating to the students’ development of a new and deeper understanding of Public Health, its implication for individuals and communities, as well as newly developed recognition of the potential impact of public health on their career focus and choice. In addition to this, four overarching themes relating to teaching and learning factors provided insight into a range of evaluative processes engaged in by the students, resulting in identification of potential areas for personal and academic improvement, as well as an appraisal of the methods utilized in the TIPH assessment task. A conceptual framework was developed to reflect the outcomes of the thematic analysis (Figure 2).

### Public Health Themes

#### New Understanding of Public Health

As expected, the strongest and most important theme to arise from the student reflections was the deep insight that the students had gained of the complex and ubiquitous nature of public health. This deeper understanding of public health, the wide scope of public health interventions, and the recognition of its impact appeared to have been grounded in a process of self-discovery facilitated through the TIPH task. For some, this had presented a powerful new perspective;

As I progressed through the assignment … I began to understand what public health is more clearly and I feel like I am developing a whole new perspective on the world (CT, 1st year medical radiation science student).

Almost all students expressed having had such an instantaneous, and often overwhelming, “light bulb” moment in which they recognized the enormity and ubiquity of public health;

As I researched more into what was ‘public health’, I found it was all around me and that was really eye opening for me (SG, 1st year occupational therapy student).

For most students, this new view had replaced a perception of public health as being limited to hospitals and medical centers;

My impression of Public Health had been of hospitals treating sick patients but this was soon challenged as I discovered more examples were actually out in the community. I began to see that Public Health is all around us (GS, 1st year Nutrition and Dietetics student).

Many students had similarly gained an eye-opening recognition and appreciation of public health interventions and strategies in place and their importance to the health of individuals and communities; many of which had previously been taken for granted, with one student noting that the task had “…*in essence changed the way [he] perceived the health of a population*.”

I began to realise the extent to which public health issues encapsulate most aspects of my lifestyle and those around me. Whether it be my habitual hygiene practices and my knowledge of their purpose, or the unpolluted air I inhale, assured of its safety, I was swiftly made to acknowledge my own ignorance in taking such aspects for granted (BN, 1st year psychology student).

With these newfound insights also came, for many students, an awareness of intervention gaps and existing health disparities between populations nationally and globally. Many students indeed showed evidence of an emerging understanding of the complex causes of poor public health outcomes in disadvantaged populations. For most students, the task had fostered a belief in the ability of public health to affect major changes to the health of individuals and communities, which for many translated into a new appreciation of the importance of preventative population-wide measures;

Since completing this assessment I have become a strong believer that public health has the potential to completely change our populations’ health (RG, 1st year occupational therapy student).

For quite a few students, this also went hand-in-hand with a greater awareness of the impact of lifestyle choices and the public health obligations of organizations and individuals;

I am now more aware and believe that every individual is responsible for health not only for themselves but in their home, community and nation. I hope to put this knowledge and experience to use and become more active in promoting the public health initiatives that are in place and advocate further improvement and future campaigns (PS, 1st year physiotherapy student).

Other aspects related to students’ understanding of and interaction with public health included an improved understanding of the complex interdependency of public health and other disciplines and a commitment to more actively contribute to, and advocate for, public health in their future roles as health professionals.

#### Student Selection of Public Health Issues

Few students reported having thoroughly researched their chosen public health focus areas prior to commencing photographing. Most proceeded to take their photographs after some brief research to gain a better understanding of public health in order to generate ideas for the 10 issues. In many cases, the final choice of focus areas were selected according to which had the most easily accessible and reliable resources, and many students found that they had to reevaluate their selections due to being unable to locate sufficient academic references, or findings that their initially selected topics had previously been used for the assessment and already had been “tagged”. However, some students had found this to be positive prompt to explore further and less obvious examples of public health and “thinking outside the square.”

Many students had felt compelled to look at examples in their local areas or issues that affected them personally, or topics that they felt were of particular current significance, giving the task a personal meaning;

I choose air pollution and smoking cigarettes topics because I am easily affected by low quality of air. I have had asthma since I was three years old. So, all my life I have been alert and conscious about air quality (MN, 1st year biomedical science student).

Many students commented on their surprise at recognizing just how prevalent public health initiatives were in their local environs and the extent of strategies put in place to protect and improve the health of local communities.

It was unusual to view the town from a different perspective, as I had never thought about ‘real’ public health before (LP, 1st year occupational therapy student).

#### Meaning Attached to Students’ Interaction with the Community

Most students had some interaction with the public as part of the task, with few students actively avoiding attracting attention. This “public display” in most cases initially was met with some trepidation due to an anticipated negative reaction to the act of placing stickers on public property. Many students reported having had an unexpectedly positive encounter with members of the public and thoroughly having enjoyed imparting their new knowledge of public health. Others reported simply having had curious glances cast at their activity. In quite a few of these cases, this had left some regret over not having had the opportunity to explain, discuss, and educate;

I went to public places and people were looking at me whilst I was doing this. They looked curious and interested. Some even stopped and asked what I was doing. I explained to them about the campaign … I was feeling proud that I could explain to them what the campaign was about due to my knowledge and research on the campaign beforehand. Everyone gave me positive responses. I felt like a professional (GB, 1st year occupational therapy student).

One of the most prevalent themes coming out of the student reflections centered around the esteem attached to their interaction with the public, as well as the learning which this interaction had facilitated. In almost all instances, the students had felt proud of their contribution to educating the community, with a few expressing their disappointment in learning of the lack of awareness and appreciation of public health among the public.

Explaining the campaign and their purpose to others clearly helped many gain more clarity on the meaning of public health, and for some students helped to highlight areas which they lacked knowledge of;

I was glad that others were asking questions about [the] campaign, explaining my task to people helped me understand it more (PL, 1st year medical radiation science student).

One of the most frequently mentioned benefits mentioned by students was the interaction with the public which the task had afforded. Not only was this additional dimension to the task perceived to broaden their understanding of public health issues but also regarded as an important avenue for educating the public;

I think the task was effective in not only educating me personally and getting me out and involved in the community, but also in creating awareness for the community about what exactly is public health (KM, 1st year physiotherapy student).

However, some students expressed regret for not having sufficiently researched the area prior to facing interaction with the public during the photo shoots, which they felt would have enabled them to speak more confidently about public health with those interested members of the public they had encountered. Some students revealed they would have liked to have taken a more active role interacting with the public to advocate for the campaign and increase awareness of public health. This appears to have been related to feeling more knowledgeable and, therefore, better equipped to discuss the subject in a more authoritative manner.

In addition to wishing to increase the impact on their community and raising the profile of public health initiatives (both of which transcends their immediate personal interests), some students also felt that their own learning could have been enhanced by greater interaction with the public. They felt that the perspectives of the community and other health professionals may have facilitated a deeper understanding of public health. Finally, another frequently mentioned reflection was the notion of complexity. Students felt that many of the issues they photographed were “easy” or “common” and were then more aware of discovering and seeking the less obvious “harder” issues that were more complex such as health issues impacting those living in rural areas or Aboriginal Australians.

A lot of people did topics like smoking and skin cancer etc which were easy to find and photograph. I did Aboriginal health as I really don’t think enough people know the real situation. I thought I had a good understanding from high school but when you are actually in a community trying to find an intervention to photograph it was really hard. It seemed invisible. I think this task could be just on Aboriginal Health for maximum impact (JH 3rd year psychology student).

#### Reflection on Public Health in Australia

Involvement in the TIPH sticker campaign left many students with a deeper appreciation for the Australian health-care system and a sound understanding of how access to basic services and resources (such as clean water, tobacco legislation, and water fluoridation) have a profound impact on the health of Australians, more so than medical/curative care. This left many students commenting on the fortunate position of Australians in comparison to those living in less developed nations. On the other hand, many students had also, as a result of their research for the task, been able to identify some of the health inequalities that exist in Australia, with urban/rural and Indigenous issues particular mentioned. One international student expressed shock and disbelief at the plight of Aboriginal Australians;

I still cannot understand how these people (Aboriginal Australians) have such poor health outcomes with little improvement in life expectancy when so much available [sic] to improve the social conditions. The more I read the angrier I be (sic). Coming from an overseas perspective I feel that learning the difference between equality and equity should be a priority for non-Aboriginal Australians to understand (AM, 1st year podiatry student).

One of the main findings was the adoption of a broader “big picture” view of public health, which revealed an appreciation of the manner in which research and local policy link together to inform and improve the health of Australian communities. However, with this realization also came, for some students, a recognition of the need to reorient efforts away from treatment-focused health care to population-based prevention strategies.

If we know that better health outcomes is really based around sensible community-wide spending to prevent ill health in the first instance, why aren’t they doing it? It seems so simple and people don’t seem to be informed of this (CH, 1st year physiotherapy student).

Indeed, for a few students, these new insights led to frustration at the lack of efforts to target disease burdens so easily addressed by prevention and of the general perceived ignorance of the public. Similarly, there was evidence of an emerging realization of the sheer extent of some of the major public health challenges in Australia such as smoking and obesity, in terms of both government expenditure and disease burden. Many students also commented on specific public health issues which they had encountered during their research for the assignment, with some relaying their surprise at gaps which they identified in public health service provision or the public’s apparent disregard for the importance of such initiatives. As a result, many expressed frustrations targeted at the lack of action taken, while others had felt compelled and inspired to extend their knowledge of the identified issues;

My new-found knowledge inspired me to want to learn more about so many things that I had taken for granted, such as, who decided to build shelters over the playgrounds? Who decided to make busy Darby St into a 40km/hr zone? (ML, 1st year Nutrition and Dietetics student).

In many instances, there was evidence of deeper learning, as students delved into the more complex interactions and relationships that underpin public health efforts;

My only concern with the concept of public health is: how do we respect the rights of the individual who may not want to vaccinate or take medication, even though there are obvious benefits to the wider population if they do so? (FH, 3rd year biomedical science student).

#### Embodying the Educated Citizen Philosophy

It was apparent from the reflections that completion of the task had had an impact on a personal level for a lot of the students, beyond those related to improved knowledge and academic performance. More than half of the students alluded to an increased appreciation of their own health (i.e., not taking one’s own good health for granted) and a new recognition of the importance of preventative health measures put in place to obtain and maintain the health of themselves and their local communities;

It made me feel much more aware and appreciative of things that have been done to protect my own health (MK, 1st year occupational therapy student).

Many students also reported having developed an improved recognition of the consequences of lifestyle choices, and a subsequent appreciation of the importance of public health awareness in minimizing the impact of poor lifestyle choices, with many relating these “discoveries” back to their own circle of family and friends. Other students had viewed the public health assessment task as a catalyst for further building their knowledge of public health fueled by a heightened interest in, and passion for, public health issues. This was often expressed as a determination to stay abreast of new public health developments.

I now endeavor to delve further into certain aspects of public health on my own and continue building my knowledge on such a vital and interesting issue for our world (MZ, 1st year medical radiation science student).

#### Public Health Workforce Implications

For many students, the task renewed their determination and passion to pursue a career in allied health. For many, this was sparked by their new-found knowledge of public health priority areas and health statistics or, in some cases, realization of the role they potentially could play as a health advocate when coming face to face with public health issues in their local communities; a role which many students enthusiastically embraced. The student reflections on the task also indicate that it helped students develop a broader view of public health and hence a realization of a more diverse range of career opportunities. Quite a few students indeed commented that the task had sparked an interest in pursuing a career specializing in public health. The task similarly seemed to have been valuable for students in that it made the links between their own discipline and public health more tangible; links which previously had not been well established for most students. A few students commented that their involvement in the task had helped give them a more holistic view of health and a broader understanding of how health issues can be tackled, which they felt would benefit their interaction with, and treatment of, future clients within their chosen field of study and would enable them to adopt a broader context through which to approach the health of their future clients/patients. Many students gave examples of how they could envision an integration of public health practices into their (future) clinical practice, with most perceiving it of great importance to incorporate a holistic view grounded in disease prevention;

Especially in my area of physiotherapy, understanding preventative actions and public health beyond medical care is extremely important in order to give a patient the best possible care and to understand what we as health professionals will be working towards; a preventative approach (LN, 1st year physiotherapy student).

Tied in with this broader view of public health, students had in many instances also come to adopt a more multidisciplinary view of allied health encouraging them to take a more integrated view of their disciplines and potentially prevent them from viewing their profession in isolation from other related disciplines, but rather as an important component in the system working to achieve and maintain the health of individuals and communities;

The TIPH campaign not only raises awareness in our communities but gives public health students a more well-rounded, comprehensive understanding of how to ensure good health beyond their own discipline (PL, 1st year physiotherapy student).

### Pedagogy Themes

#### Initial Perceptions of the TIPH Experiential Task Stimulates Interest

Approximately half of the students expressed feelings of excitement when they were first informed of the TIPH photo essay and reflection task. Many said they felt enthusiastic simply due to the assessment task presenting a pleasant variation from the usual essays and allowed integration of creativity and the use of other technologies (i.e., photography). For many, however, this initial enthusiasm in the task faded together with a growing perception of the enormity of the task. This concern was largely about not being able to “find” 10 examples of public health interventions that they could photograph for the task.

I was really excited about taking photos for an assessment task like this until I realized I wasn’t going to be able to find ten things to photograph. By the time I finished the task and then the course I realized it was everywhere in every part of where I work and live. I felt rather silly thinking back at my initial panic and realized why they made us do the task. I think it’s a great idea (JD, Year 1 Medical Radiation Science student).

The remainder of students expressed feelings of apprehension due to uncertainty about their understanding of the concept of public health, what it constitutes, and subsequent difficulties in identifying issues outside of the formal health-care system. A small number of students had similarly felt quite skeptical as to how the task, and public health *per se*, would relate to their degree and hence questioned what it would achieve. Some students had also felt considerable reluctance toward the task due to suspected negative reactions of the public toward placing of stickers on public property (i.e., fear of allegations of vandalism). This led students to solicit the assistance of friends or family to accompany them or choose locations away from the public eye.

#### Challenges in Finding and Using Evidence to Support PH Issue Selection

Most students experienced difficulty differentiating valid evidence sources. Finding and using evidence to support ideas posed a challenge. However, finding and using evidence for this public health task was first related to a reluctance and/or concern about accessing contemporary, reputable “web site” resources from governing bodies and/or authorities. Students reflected that in their other courses they were generally discouraged from using “web site” resources in preference for journal articles. Second, students reported difficulties locating relevant legislation or policy documents underpinning most public health interventions.

I have never had an assignment where we were encouraged to use websites to find information and it seemed like a trick. So I started looking at NEWCAT (library database) and there was nothing on the laws or policies that we used in the photos. Actually there was nothing really concrete in any of the journal articles I found that addressed the marking criteria. I then started to really panic. When I got help from the course coordinator who took me through the process of working out which websites were valid and reliable I was really excited to research all my photos There is so much important and up to date information on government and non-government authority websites for health issues - we just had to learn how to tell them apart (from poor quality sites) (JB, 1st year physiotherapy student).

In contrast, some students commented that they had been overwhelmed by the large amount of information available making it difficult to use this evidence, synthesize the information, and present their argument in a brief and succinct manner while maintaining focus in their writing. In their reflection, many students contributed their writing difficulties to having chosen their focus areas and taken their photos prior to having researched the areas, leaving this component of the task until last, often resulting in the need to modify their approach.

I felt like I was drowning in information and I didn’t feel confident in assessing the most important information to put in. It all seemed important to me. There was just so many different statistics and reports that I couldn’t work out what to use and what not to use. There was too much information to cover the things in the marking criteria in such a small word count and I was really stressed (AG, 1st year nutrition and dietetics student).

#### Task Acceptability and Student Engagement

Overall, the TIPH photo essay and reflection task was very well received by students. It was perceived as particularly inventive, creative, and enjoyable and had appealed to almost all students. Not only was it felt to facilitate enhanced learning through a practical and hands-on approach, it was also, by some students, described as a unique opportunity for personal growth and tailored learning, due to the personal choice afforded by the task. With the learning method, clearly different from standard assessment tasks, students found themselves face to face with real-life issues in the community which, for many, gave the task more personal meaning and facilitated greater engagement;

I can honestly say that the instantaneous real world application of this task is beyond any I have previously undertaken (BB 2nd year Biomedical Science student).

Similarly, many students also commented on the hands-on approach having facilitated a greater understanding and appreciation of the preventative measures in place to protect the community. This was also achieved by student’s perception of their great personal involvement and ownership of the campaign, which was expressed eloquently by one student;

We weren’t just researching Public Health awareness; we were becoming a part of it (MCD, 1st year occupational therapy student).

## Discussion

The thematic analysis presented here aimed to evaluate the role of the interactive TIPH photo essay and reflection task in improving and deepening undergraduate students’ understanding of public health and awareness of its breadth of meaning. In addition, the analysis aimed to explore the subjective experience of gaining this understanding. The analysis yielded two distinct thematic pathways. The first pathway was related to public health knowledge development in which there were six overarching themes; new understanding of Public Health was related to students having to select their own public Health issues, interacting with the community and subsequently reflecting on Public Health in Australia. Students embodied the “Educated Citizen” philosophy identifying the intrinsic role public health has across all societies. The realization of workforce implications beyond health professionals was also evident. The second pathway was related to pedagogy with three overarching themes related to teaching and learning. Initial perceptions of the TIPH experiential strongly stimulated interest across the cohort. The challenging cycles of finding and using evidence to support the public health issues they selected were also strong factors contributing to the process of increased knowledge. High task acceptability and student engagement were evident in student reflection. In summary, students do gain a new and deeper understanding of public health during the process of completing the TIPH photo essay and reflective task, and the pedagogy behind the task has an intrinsic role in this process. The TIPH photo essay activity was successful in engaging students and increasing their understanding of public health. The interactive nature of the experiential TIPH task led to “light bulb” moments in depth of understanding and the reflective component of the task was very important in this process.

Public health education aims to address the definitive need to ensure that our future leaders are contributing members to local, national, and global communities, and to be able to apply their acquired knowledge to real-world problems. In addition to discipline specific knowledge, skills, and attitudes (23), the different purpose of 21st century education requires the new generations of graduates to be competent in self-directed learning, citizenship, eco-sustainability, and employability in response to emerging global challenges. Embedding experiential learning into undergraduate programs is especially crucial in public health teaching and learning, and this analysis demonstrated that using the TIPH task was an effective tool to provide students with an opportunity to reflect on becoming educated citizens. Once students had moved beyond a text book or literature definition of public health and actually physically had to go out into the community, they realized that public health was absolutely everywhere and beyond their expectations. These consistent narratives were frequently identified in the reflective part of the task and enabled the “light bulb” moments to happen in response to the experiential activity.

An intrinsic part of the experiential learning is the ongoing cycle described by Kolb earlier. Reflection is a key part of this process and provides an interface of understanding or “light bulb” moments, which are required to facilitate deeper learning (24). The link between the theory and “real life” can be lost without a student’s ability to reflect on their experience. In this paper, the reflections enabled a deep exploration of student experience during the assessment task. The nature of such a reflective piece illustrates “… *different points of time and to move between the past, present and future* …” (25). Timing is intrinsic for students reflections in this task. Students were required to analyse their own learning experience and knowledge transition during the completion of the task (17). This is relevant when considering the number of photos required for the experiential activity as more examples mean more time and hence more opportunities to reflect.

This study has several strengths. There are very few published evaluations utilizing the TIPH activity in tertiary teaching and assessment. Indeed, there are few rigorous evaluations of undergraduate public health pedagogy overall (26). Evashwick et al. (26) identified only three detailed assessments of undergraduate public health teaching techniques in the USA in their literature review from 2004 to 2014. Unlike one previous study (19) that focused on one specific topic, the current study allowed for a broad experience requiring students to identify and photograph 10 areas of public health. The current study is also the first to include a reflection activity alongside the photo essay (which represents best practice in experiential learning in tertiary education). The qualitative analysis was conducted by two authors. However, the study does have some limitations that should be taken into consideration when interpreting the findings. First, the study was only conducted in one university, utilizing a single cohort of students over a single semester. Second, in the current study we analyzed only the student reflections of the task, in contrast to Ref. (4) who analyzed photo content.

Despite the number of academics involved in teaching undergraduate public health and the indicated uptake of this campaign in the USA alone, “*few have written for the professional literature indexed in PubMed, Scopus, or ERIC”* (9). Subsequently, there are few formal evaluations of the TIPH photo essay task in the tertiary setting to compare our findings to. Consistent with the findings in our study, the TIPH task evaluated by Ref. (19) resulted in new and deeper understanding of public health. Chase et al. focused on individuals’ recycling behaviors and reported that participating in a range of TIPH campaign events over a 1-month period resulted in an increase in self-reported recycling behaviors among both students and community members. In addition, an unexpected outcome was the uptake of the campaign by other areas of the university, the wider community, media agencies, and the government. Partnerships developed secondary to this increased exposure and consumption of the TIPH campaign and brand with additional recycling opportunities. These results are consistent with uptake and use of the TIPH campaign that is widespread in the USA and internationally. In contrast with our findings and those of Chase et al. (19), Geraghty and Fanany (4) concluded that students did not gain increased understanding of public by completing the photo essay. However, students in the Geraghty and Fanany study identified and photographed just three areas of public health and the authors predominantly analyzed only the content of the three photos. The authors concluded that the task resulted in limited knowledge of breadth and scope of public health. This finding may differ from the current study due to the task having reduced scope (three photos compared to 10, no reflection task, and no requirement to describe the public health issue and intervention) or the focus of the analysis on the photo content alone (4). Further use, satisfaction, or uptake of the campaign was not discussed.

Despite the lack of published empirical evaluations of the TIPH campaign, the ASPH have such belief in the campaign that they have made it the centerpiece of their online marketing efforts. Their rationale explained in a report in 2009 *“The growth of the program clearly indicates that in the eyes of its users, the TIPH program works well”* [(18), p.3]. The report of the first 12 months of the TIPH campaign evaluation released in September 2009 revealed the TIPH website had received over 1.3 million hits and visited 72,439 times by over 43,000 unique visitors. The campaign hashtags #TIPH and #ThisIsPublicHealth were incorporated to track submissions across social platforms. Presently, there are millions of TIPH campaign photos with the abovementioned hashtags on Flickr; hundreds of Facebook, Instagram, and Twitter pages dedicated to multiple organizations and schools who have implemented the campaign and documented their experiences on social media. The 2009 document also reports on phone interviews, e-mail communication, and surveys with program participants and feedback from a number of public health groups. Six hundred people (20% responses rate) who utilized the campaign were surveyed to explore how participants used the campaign and their perceived strengths and weaknesses. The majority of respondents [92%] said that they were likely to use the campaign again and recommend it to colleagues in the public health community (18).

It appears that international uptake, use, and satisfaction of the TIPH campaign are high, with a significant presence evident on social media. However, from an empirical perspective it is difficult to demonstrate the effectiveness of an undergraduate public health education activity (TIPH campaign) based on uptake, satisfaction, and social media presence alone. Social media campaigns successfully improve reach and user engagement but data remain largely anecdotal (27). While Evashwick et al. (26) flag the gap in peer reviewed literature about undergraduate public health pedagogy, they acknowledge other indicators such as increased information sharing *via* other alternative online sources such as blogs, Listservs, websites, and social media (26). Popular BLOGS such as “POP health: examining the intersection of Public Health and Pop Culture” are starting a dialog about addressing this evidence gap. However, this dialog is occurring within a blog rather than traditional methods that demonstrate *the status of a field of study* that peer reviewed literature does (26). The TIPH campaign most definitely demonstrates the use of pop culture to convey messages. Another difficulty in evaluating the effectiveness of an activity such as the TIPH campaign is that it is used by a wide variety of consumers in a multitude of ways. Despite this, and the gap in formal evaluations, The ASPH (28) say that the TIPH campaign *“has ‘proven’ to be a powerful tool in raising awareness about and support for public health”* (28). They go on to say that strategies used in the TIPH campaign are crucial in an era when the public’s time and attention are increasingly directed toward social media sites. While use of the internet and social media is intrinsic to young people for opportunities to empower themselves (29), the question remains whether it is appropriate to rely on such information to replace empirical evidence.

### Recommendations and Conclusion

The TIPH photo essay and reflection was well accepted by undergraduate students, feasible to conduct and resulted in a new and deeper understanding of public health. University educators across multiple disciplines should consider use of the TIPH photo essay and reflective task as a teaching and learning activity and assessment task. More published evaluations documenting the impact of TIPH activities will add to the body of knowledge in teaching techniques in undergraduate public health education. TIPH photo essays or activities should include at least 10 photos to ensure that the broad scope of public health is realized by undergraduate public health students. These photos and/or subsequent activities should also have a requirement to document the associated health issue. Reflection is an essential element of deep learning and hence is a valuable component of experiential learning tasks. Future evaluations of the TIPH photo task may consider analysis of the content of photos and/or add additional experiential elements.

## Ethics Statement

This study was carried out in accordance with the recommendations of the University of Newcastle Human Research Ethics Committee (H-2010-0024). All subjects gave written informed consent in accordance with the Declaration of Helsinki. The protocol was approved by the University of Newcastle Human Research Ethics Committee (H-2010-0024).

## Author Contributions

EJ, SO, and KD conceived the study and obtained ethical approval for its conduct. KD recruited participants. VH and KD conducted the analysis. All the authors contributed to the interpretation of data, drafting the manuscript, and revising it critically for important intellectual content. All the authors approved the final version to be published and take responsibility for the accuracy and integrity of the work.

## Conflict of Interest Statement

All authors declare that the research was conducted in the absence of any commercial or financial relationships that could be construed as a potential conflict of interest.
